# Mechanistic Insights into Oxidative Stress and Apoptosis Mediated by Tannic Acid in Human Liver Hepatocellular Carcinoma Cells

**DOI:** 10.3390/ijms20246145

**Published:** 2019-12-05

**Authors:** Priscilla Mhlanga, Pearl O. Perumal, Anou M. Somboro, Daniel G. Amoako, Hezekiel M. Khumalo, Rene B. Khan

**Affiliations:** 1 Discipline of Medical Biochemistry, School of Laboratory Medicine and Medical Science, University of KwaZulu-Natal, Durban 4000, South Africa; pltshoaedi@gmail.com (P.M.); eleiro.pop@gmail.com (P.O.P.); kumaloh@ukzn.ac.za (H.M.K.); 2 Biomedical Resource Unit, School of Laboratory Medicine and Medical Sciences, College of Health Sciences, University of KwaZulu-Natal, Durban 4000, South Africa; anou.somboro@gmail.com

**Keywords:** tannic acid, apoptosis, oxidative stress, ROS, HepG2 cells

## Abstract

The study investigated the cytotoxic effect of a natural polyphenolic compound Tannic acid (TA) on human liver hepatocellular carcinoma (HepG2) cells and elucidated the possible mechanisms that lead to apoptosis and oxidative stress HepG2 cell. The HepG2 cells were treated with TA for 24 h and various assays were conducted to determine whether TA could induce cell death and oxidative stress. The cell viability assay was used to determine the half maximal inhibitory concentration (IC_50_), caspase activity and cellular ATP were determined by luminometry. Microscopy was employed to determine deoxyribonucleic acid (DNA) integrity, while thiobarbituric acid (TBARS) and nitric oxide synthase (NOS) assays were used to elucidate cellular reactive oxygen species (ROS) and reactive nitrogen species (RNS), respectively. Western blotting was used to confirm protein expression. The results revealed that tannic acid induced caspase activation and increased the presence of cellular ROS and RNS, while downregulating antioxidant expression. Tannic acid also showed increased cell death and increased DNA fragmentation. In conclusion, TA was able to induce apoptosis by DNA fragmentation via caspase-dependent and caspase-independent mechanism. It was also able to induce oxidative stress, consequently contributing to cell death.

## 1. Introduction

Cancer is one of the leading causes of death worldwide with a high mortality rate, which makes it an imperative public health issue [1]. Environmental and lifestyle factors, including smoking, diet, and lack of exercise, cause 90% of all cancers. Liver cancer has a steadily increasing mortality rate at 8.2% of all cancer-caused death worldwide [2]. It has a poor prognosis of about 31% for people with localised liver cancer from different histological groups. Liver cancer ranges from hepatocellular carcinoma (HCC) and intrahepatic cholangiocarcinoma (iCCA) to mixed hepatocellular cholangiocarcinoma (HCCCCA), fibrolamellar HCC (FLC), and the paediatric neoplasm hepatoblastoma [3,4]. The most common primary liver cancers are HCC and iCCA, while others, such as mixed HCC-CCA tumor, account for less than 1% [5].

The treatment of diagnosed cancers involve surgery to remove the tumour cells, radiation to kill cancer cells using targeted high-energy rays, and chemotherapy to treat fast-growing cancer cells using drugs [6]. These treatments are often used in combination with each other. Although targeted radiation can also damage the surrounding normal cells, chemotherapy is far more damaging to the normal fast-growing cells in the body, as it is not a target-specific treatment. This results in adverse reactions in patients, causing hair loss, damaged skin cells, and intestinal cells, and their bodies being poisoned by the treatment [7,8]. The use of naturally occurring plants and their extracts that show medicinal properties are popular in the scientific community [9], however there is the need to investigate and gain insights into their mechanistic pathways.

The polyphenol molecule, tannic acid (TA), is an edible plant that is found in green tea, grapes and many other fruit plants [10]. TA is a glucoside comprising gallic acid polymers that form part of the hydrolysable tannins found to possess anti-oxidative properties that may contribute to their cancer preventive, chemotherapeutic, and anti-inflammatory properties [11,12,13]. In vivo, TA and other green tea polyphenols inhibited the mammary tumour virus promoter, whereas in vitro the proliferation of specific cancer cell lines was inhibited and induced apoptosis [14]. TA has been revealed to exert a significant liver-protective effects in mice with CCl4-induced liver fibrosis [15]. Moreover, its chemoprotective effects against neoplasm development [16], as well as in hepatoma bearing C3H male mice [17], has also been reported. Contrastingly, the toxicity of TA has also been receiving considerable attention. The cytotoxic effects of TA have been reported in liver by Zhu et al. [18] and Korpassy [19]. However, the mechanistic pathway of TA against liver cancer in humans remains unclear; therefore, this study aimed to assess the effects of TA on liver cancer cells and the pathways that contribute TA’s oxidative stress and apoptosis effect in liver cancer.

## 2. Results

### 2.1. MTT Assay

**Figure 1:**
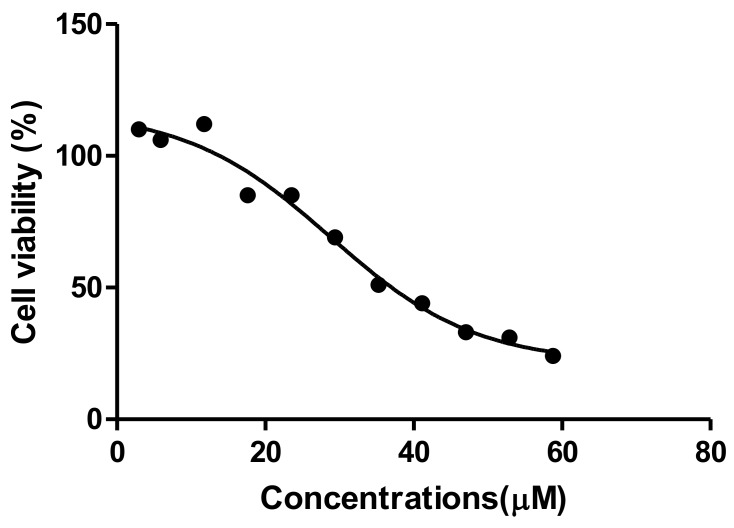
Tannic Acid (TA) decreased cell viability of HepG2 cells in a dose-dependent manner after a 24 h treatment. The data is presented as a percentage of the viable cells relative to the untreated control. An increase in TA concentration showed lower cell viability and increased death.

The 3-(4,5-dimethyl-2-thiazolyl)-2,5-diphenyl-2H-tetrazolium bromide (MTT) assay was used to measure TA toxicity after a 24 h of exposure to human liver hepatocellular carcinoma (HepG2) cells; this was to provide a preliminary data showing that TA could inhibit the metabolic activity and respiratory potential of the liver HepG2 cells at the concentrations used. Cell viability of HepG2 cells was quantified and was found to have decreased from 110% to 24% when treated with TA at concentrations of 0–58.8 µM, decreasing as the dose increased, as seen in Figure 1. The analysis showed that a concentration of 29.4 µM was sufficient for killing 50% of the cells. This concentration (IC_50_ = 29.4 µM), as well as IC_25_ (14.7 µM), were used in all the upcoming assays to understand the pathways that contribute TA’s oxidative stress and apoptosis effect in liver cancer.

### 2.2. Measuring Apoptotic Activity

**Figure 2:**
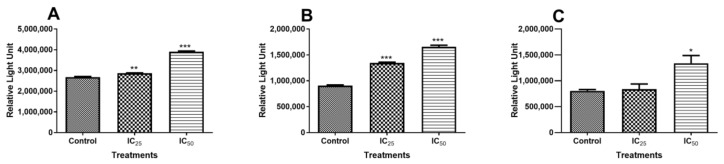
Graphical presentation of Tannic acid influence on caspase activity. **A**. Caspase 8 activity showed a significant increase and a linear relationship between TA concentration and the activity detected by the luminometry assay. **B**. Caspase 9 activity is increased in a dose-dependent manner. **C**. Caspase-3/-7 activity significantly increased in relation to the control. (* *p* < 0.05, ** *p* < 0.001, *** *p* < 0.0001, using ANOVA and student t-tests).

Luminometry was used to measure apoptotic activity in the cells caused by TA treatment of cells at different concentrations. The activities of caspase-3/-7, caspase 8, caspase 9, and the presence of intracellular ATP were analysed. The activity of caspase 8, an extrinsic apoptotic pathway initiator caspase, was increased in a dose-dependent manner. Figure 2A shows a significant increase in activity, IC_25_ has a 1.07-fold change (2,869,000 ± 9313 RLU, *p* = 0.0021) and IC_50_ has a 1.46-fold change (3,907,000 ± 17,550 RLU, *p* < 0.0001) relative to control (2,680,000 ± 16,160 RLU). An increased dose resulted in amplified activity.

Caspase 9 is an initiator caspase in the intrinsic pathway of apoptosis. As shown in Figure 2B, caspase 9 activity was significantly increased (*p* < 0.0001) by a low dose of TA (14.7 µM) and (*p* = 0.0007) by IC_50_ (29.4 µM) relative to the control. Figure 2C, below, shows the activity of caspase 3/7 after treatment with TA, the graph indicates a nonsignificant increase (*p* = 0.6124) in caspase activity in cells that were treated with the lowest dose of TA compared to the control. There was a significant increase (*p* = 0.0261) in caspase activity in the cells that were treated with IC_50_ relative to the control.

### 2.3. Measuring Cellular ATP

**Figure 3:**
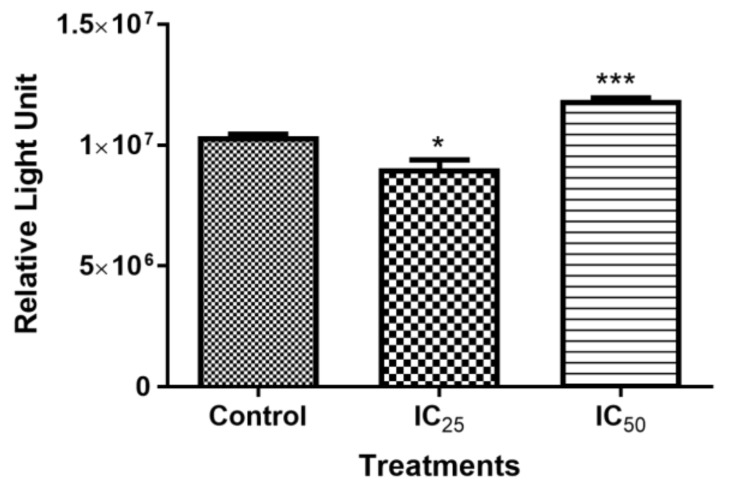
The presence of intracellular Adenosine triphosphate (ATP) after a 24 h TA treatment significantly decreased at IC_25_ and showed a significant increase at higher concentrations of IC_50_. (* *p* < 0.05, *** *p* < 0.0001 using ANOVA and student t-tests).

Intracellular ATP showed a significant decrease when the cells were treated with IC_25_ (9,047,000 ± 200,500 RLU, *p* = 0.0238) and a significant increase at IC_50_ concentration (11,860,000 ± 51,190 RLU, *p* = 0.0003), in relation to the control (10,370,000 ± 54,260 RLU), as illustrated in Figure 3.

### 2.4. Measuring Oxidative Stress

**Figure 4:**
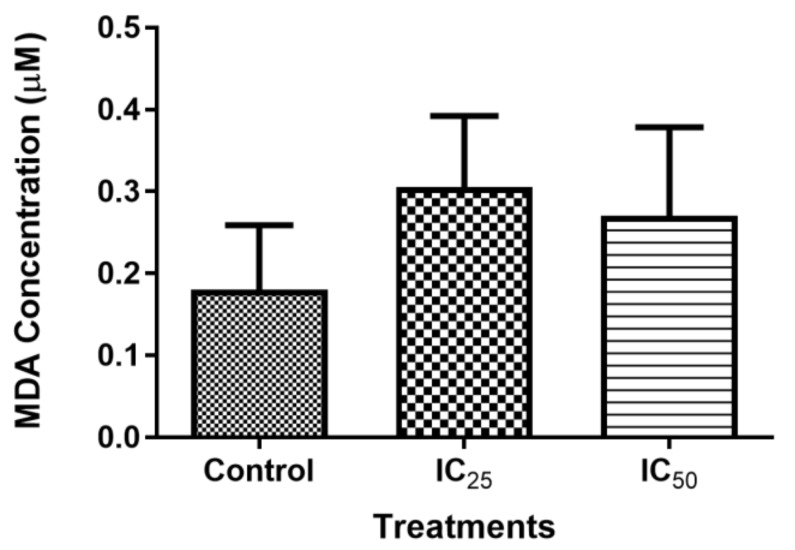
The effect of TA on intracellular reactive oxygen species (ROS) showed non-significant increase as compared to the control. (*p* > 0.05 using ANOVA and student t-tests).

**Figure 5:**
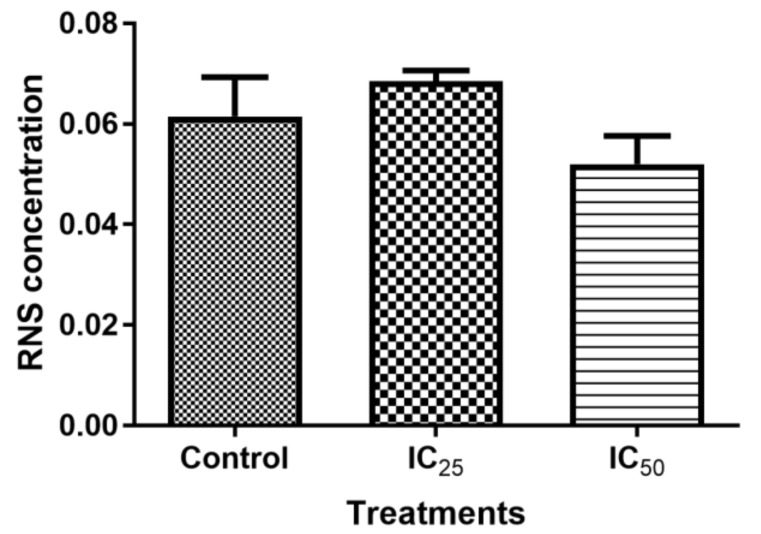
The 24-h treatment of cells by TA is illustrated to have a non-significant influence on the intracellular RNS. (*p* > 0.05 using ANOVA and student t-tests).

Reactive oxygen species (ROS) was measured while using the TBARS assay. As observed in Figure 4, ROS non-significantly increased at all the treatments, although the highest increase in ROS was found in the lowest concentration of TA. The presence of intracellular RNS after the TA treatment showed a non-significant increase in all treatments, as illustrated by Figure 5.

### 2.5. Investigating DNA Integrity

**Figure 6:**
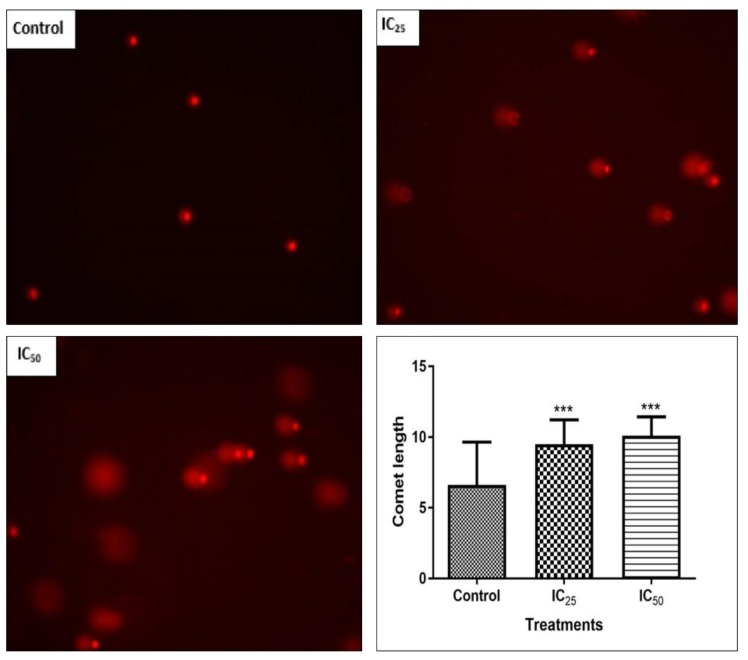
An increase in comet length after a 24 h TA treatment that is dose-dependent is observed by the comet assay (MQ × 200). This is an indication of DNA fragmentation, the longer the comet, the higher the extent of the damage in the cell. A significant increase in comet length and therefore DNA fragmentation in a non-dose dependent manner is observed. (*** *p* < 0.0001 using ANOVA and student t-tests).

**Figure 7:**
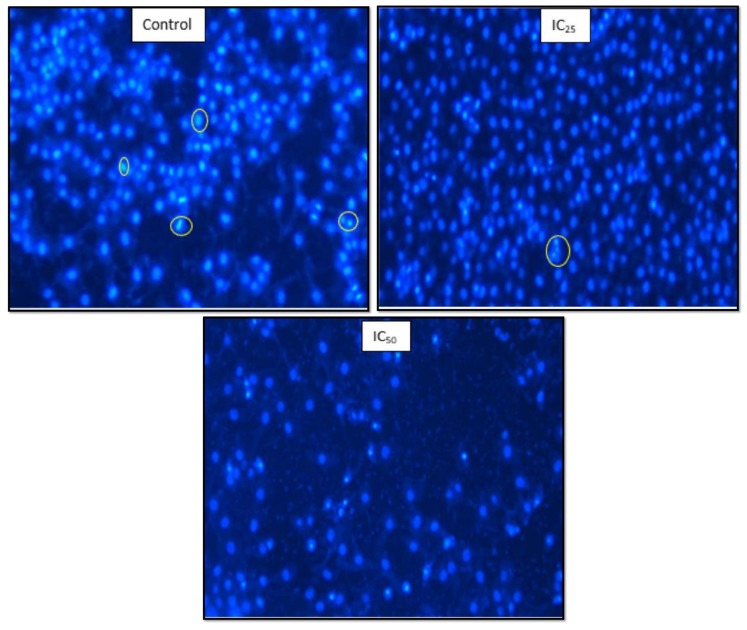
The various cell cycle stages were affected by the 24 h treatment of cells by TA (MQ × 200). There is a decrease in the number of cells going through mitosis in a dose-dependent manner and increased cell death that is inversely proportional to TA concentration.

Figure 6 is an indication of the effect of tannic acid on DNA integrity. The comets, as an indication of DNA fragmentation, were increased by the treatment. It was observed that IC_50_ has the longest comets on average, as observed. There was a significant increase (*p* < 0.0001) in the comet length. A linear relationship between the comet length and TA concentration was noted. The Hoechst assay was used to determine the effect of TA on the DNA of the cells. Figure 7 illustrates the control shows cells going through the various stages of mitosis. The mitotic activity is observed to have decreased after TA treatment. The IC_25_ contained fewer cells going through mitosis and apoptotic bodies are found, while the IC_50_ shows a decreased number of cells, with an increase in the amount of cell debris.

### 2.6. Measuring Protein Expression

**Figure 8:**
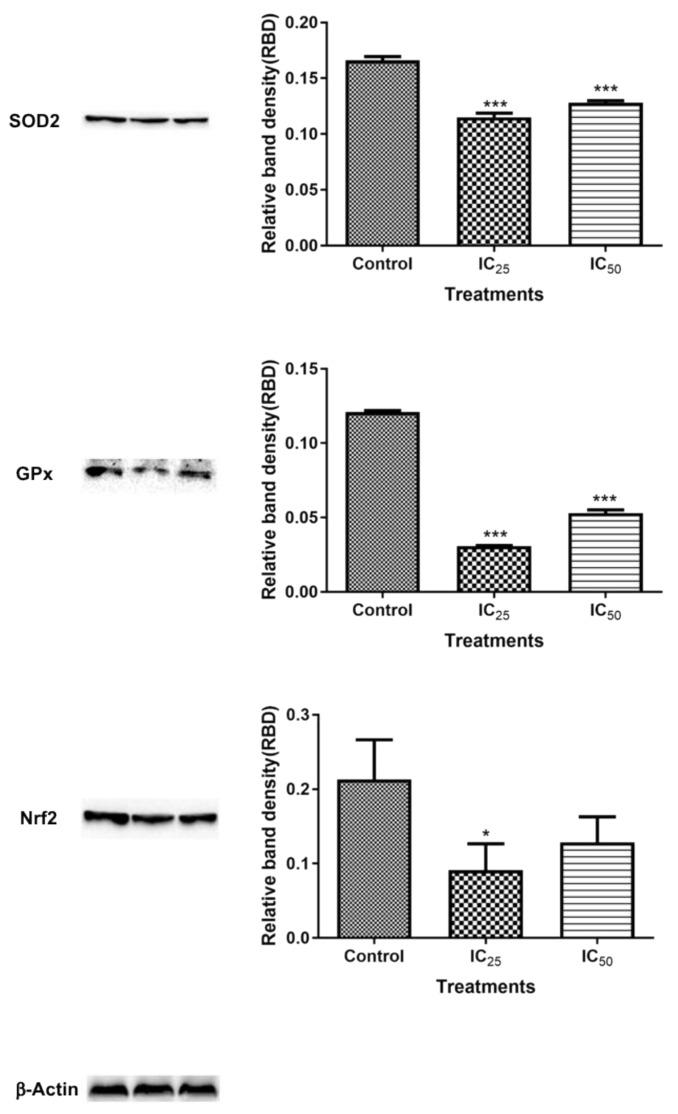
Measurements of protein expression in HepG2 cells after treatment with TA. The expression of antioxidant proteins expressions has been illustrated to be influenced by TA acid. Superoxide dismutase (SOD2) expression was significantly reduced (*** *p*< 0.001) in all treatments in relation to the control. GPx expression was significantly (*** *p* < 0.0001) decreased by TA treatment in IC_25_ and IC_50_ Nrf2 expression was significantly (* *p* < 0.05) reduced by TA treatment at IC_25_ relative to control (using ANOVA and student t-tests).

**Figure 9:**
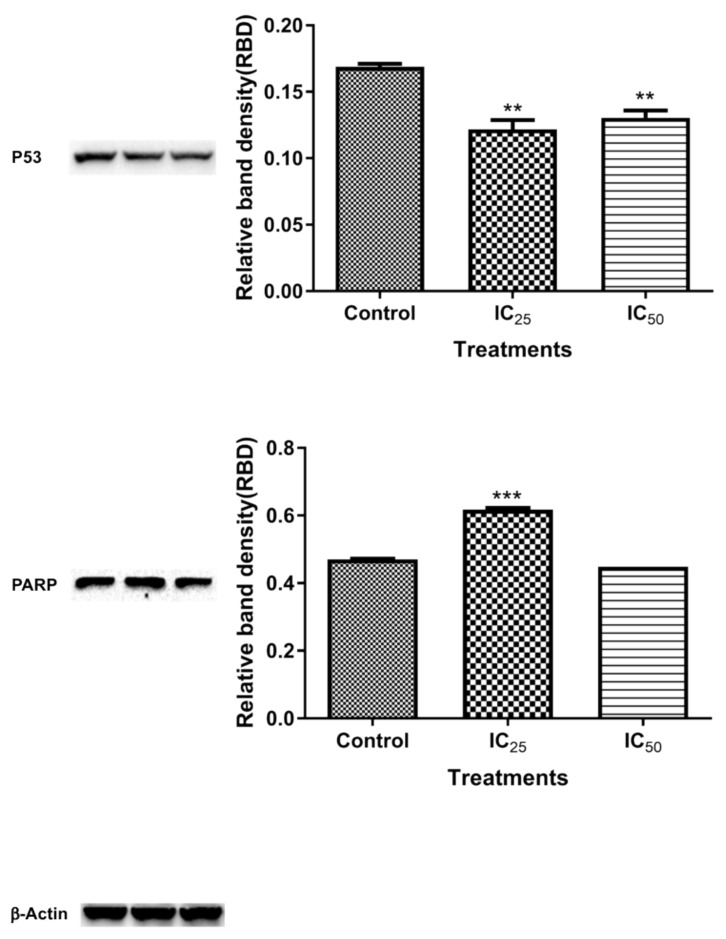
The analysis of protein expression of p53 and PARP show significant changes when compared to the controls as influenced by the TA treatment. P53 expression is significantly decreased at treatment concentration. PARP is significantly increased at the lowest concentration and a slight difference is observed between the control and IC_50_. (** *p* < 0.001 ***p < 0.0001 while using ANOVA and student t-tests).

The expression of proteins expected to increase during oxidative stress was measured and the results are analysed in Figure 8. The expression of superoxide dismutase (SOD2) (26.6 kDa) was decreased 1.44-fold (0.1151 ± 0.002076, *p* = 0.0004) at IC_25_ concentration and 1.3-fold (0.1280 ± 0.001103) at IC_50_ concentration relative to control (0.1660 ± 0.001955, *p* = 0.0004). GPx1 (23 kDa) expression decreased when the cells were treated with IC_25_ by 3.93-fold (0.03080 ± 0.0002198, *p* < 0.0001) and IC_50_ concentrations by 2.28-fold (0.05306 ± 0.001266, *p* = 0.0004), relative to control (0.1209 ± 0.0005857). Nrf2 expression was significantly decreased by TA treatment at the lowest dose (*p* = 0.0455), but at IC_50_ the decrease was not significant (*p* = 0.1033) in relation to the control. The protein expressions of p53 and poly (ADP-ribose) polymerase (PARP) are analysed and quantified in Figure 9. The p53 expression was decreased 1.39-fold (0.1217 ± 0.004090, *p* = 0.0082) at the IC_25_ treatment concentration of TA, while a 1.29-fold decrease (0.1304 ± 0.003201, *p* = 0.0080) was noted at IC_50_. On the other hand, PARP was significantly increased at the lowest concentration by 1.31-fold (0.6172 ± 0.002904, *p* = 0.0004) and there seems to be a non-significant change at IC_50_ relative to the control.

## 3. Discussion

Tannic acid (TA) is a naturally occurring polyphenolic compound that is found in edible plants, like grapes, tea, and tree barks, and they are easily accessible to the population. TA is known to have chemoprotective, anti-inflammatory, and anticarcinogenic properties. In human cell lines, like acute myeloid leukemia HL-60 cells, TA was found to induce apoptotic death as well as chromosome condensation and DNA fragmentation [9]. However, the investigation of TA on ischemic brain tissue revealed a protective effect, contrary to its effects on acute myeloid leukemia HL-60 cells [20]. This means that TA has different effects on various cells; hence, investigation of its effects and mechanisms liver cancer cells is incessant. Therefore, this study investigated the cytotoxic effect of a TA on human liver hepatocellular carcinoma (HepG2) cells and elucidated the possible mechanisms that lead to apoptosis and oxidative stress in HepG2 cell.

The MTT assay is a colorimetric assay for assessing cell metabolic activity and cellular viability [21]. Tannic acid induced a dose-dependent decrease in HepG2 cell viability (Figure 1). Decreased viability is an indication of reduced metabolic activity of cells that is caused by TA. This could be due to TA being a weak acid that disrupts the electron transport chain, subsequently reducing NADPH and rendering the mitochondrial dehydrogenase enzyme unable to perform its function [22]. This results in the observed reduction in cell viability. The reduced cell viability that was observed in the MTT corresponds to cell death, which was indicated by the IC_50_ (29.4 µM). Interestingly, Krajka-Kuzniakand co demonstrated a similar cytotoxic effect of TA against the HepG2 cells that correlated with our findings, where TA reduced cell viability [23] However, the concentration used to elucidate the parameters to describe the antioxidant effect by Krajka-Kuzniak et al. were significantly lower (2 µM) than the one that was used in our study; hence, the marked difference in the effects that were observed on the Nrf2/ARE pathway in HepG2 cells. A similar low dose administration of TA was reported to have a chemoprotective effect on hepatoma bearing C3H male mice [17]. These contrasting results indicate that the concentration of tannic acid administered plays a significant role with its effect on liver cancer cell lines.

Initiator caspases-8/-9 and executioner caspase-3/-7 activities were investigated to investigate the mechanism of cell death that resulted from TA treatment. Caspase 8 activity was not increased at the lower dose (IC_25_) of TA (Figure 2A). However, the IC_50_ dose showed increased caspase 8 activity. This is indicative of increased apoptosis initiation. It has been reported that apoptosis can be induced via the receptor-dependent, physiological activation of apoptosis, and this activation resulted in the cleavage of the procaspase-8 molecule into an active caspase-8, which is an initiator of apoptosis [24]. However, caspase 9 activity also increased with increasing TA dose (Figure 2B), indicating that the intrinsic, mitochondrial-dependent activation of apoptosis was also triggered by TA, displaying activity that is even greater than that of caspase 8, whose activity was increased, even at a low TA dose. Treatment with TA increased the initiation of apoptosis via both the receptor- and mitochondria-dependent apoptotic pathways at the IC_50_ concentration, although the intrinsic pathway was more favoured by TA. This trend in the activation of caspases has been observed in HL-60 cells, where both initiator caspases were activated and, when compared, it was observed that caspase-9 had greater activation than caspase-8 [9,25,26].

Initiator caspases can directly activate the effector caspase-3/-7 via different pathways. Active caspase-8 can cause the direct activation of caspases-3/-7 and it can cleave BH3-only protein Bid to form a truncated version, t-Bid [27]. When t-Bid translocates to the outer mitochondrial membrane (OMM), it activates BAX, a pro-apoptotic protein. Activated caspase-9 can directly convert procaspase-3/7 into the effector caspase-3/-7, which plays a leading role in effecting apoptosis [28,29]. Caspase-3/-7 was compared to the control (Figure 2C). IC_25_ showed no variation in caspase activity, which implies that there was no increase in apoptosis. However, caspase-3/-7 was significantly increased at IC_50_, thus increasing apoptosis.

DNA fragmentation, reduced cell numbers, and the formation of apoptotic bodies are hallmarks of apoptosis [28], therefore Hoechst and SCGE assays were carried out to observe the effect of TA on the cellular DNA. Figure 6 confirmed the presence of DNA fragmentation on a single cell level, where an increase in TA showed longer comet tails and, therefore, a greater extent of DNA damage on the cells. The increased presence of caspase-3 activity in the cells and DNA fragmentation observed in comet assay indicate that TA is able to trigger apoptosis. A study by Chen et al. reported that TA induced apoptosis by internucleosomal DNA fragmentation, cell shrinkage, chromatin condensation, and apoptotic body formation [9]. Figure 7 showed that a low dose of TA had fewer HepG2 cells undergoing mitosis while apoptotic bodies were observed. The IC_50_ showed a low cell population and cell debris, which is an indication of cell death.

The effect of TA on cellular ATP was investigated, since apoptosis is an energy-dependent process [30]. It was observed that Adenosine triphosphate (ATP) production was affected by higher dose of TA, (IC_50_ treatment), which correlates with caspase-3/-7 activation at the concentration of TA (Figure 2C). This indicated that TA induced caspase-dependent apoptosis at high concentrations, whereas caspase-3/7 were similar to the control and ATP production decrease at a lower dose of TA indicated caspase-independent apoptosis.

Caspase-independent apoptosis might occur when changes in the mitochondrial membrane potentially occur and ROS generated in the respiratory chain gets involved [28]. Increased concentrations of ROS cause the ATP/ADP antiporter to be converted into a gigantic channel, by which the cytochrome c, apoptosis inducing factor (AIF), which is capable of activating cytosolic proteinases, and other proteins leave the mitochondria into the cytosol, resulting in the translocation of AIF and Endonuclease G into the nucleus where they take part in the caspase-independent apoptosis. AIF can directly bind to DNA and its DNase activity can cause chromatin condensation and DNA fragmentation [28]. The presence of activated caspase-9 confirmed the pore opening and release of cytochrome c, which plays an important role in the activation of caspase-9. Furthermore, the activated caspase-9 (Figure 2B) could have been inhibited by the inhibitor of apoptosis protein (IAP) that could have been released from the mitochondria with other molecules, such as cytochrome c. IAPs bind to the activated caspase-9, thus inhibiting its function of cleaving procaspase-3/7 to activate it [31].

Activated caspase-3 (Figure 2C) has two targets, it frees and activates caspase-activated DNase (CAD) from its inhibitor by cleavage. Caspase-3/7 also targets the poly (ADP-ribose) polymerase (PARP), which is a nuclear enzyme that transfers the negatively charged ADP-ribose moiety from nicotinamide adenine dinucleotide (NAD^+^) to protein substrates, participating in DNA repair by binding to the DNA site that needs repair together with DNA ligase, which hinders the separation of the broken DNA and promotes adhesion of the broken sites [31]. Figure 9 shows the expression of cleaved PARP after the TA treatment. The IC_25_ cleaved PARP is an indication of increased apoptotic cell death, due to “suicidal” proteases, like caspases, calpains, cathepsins, granzymes, and matrix metalloproteinases (MMPs) [32]. PARP cleavage is expected to be carried out by a different ICE-like caspase that was observed during in vivo studies since caspase-3/-7 was shown to have not increased at this concentration [33]. IC_50_ showed an increased presence of ATP, which is consistent with caspase-dependent apoptosis, since it has been demonstrated to be an energy-dependent process.

The mitochondria are one of the major participants in ATP production. It is also involved in cell apoptosis, as changes in mitochondrial membrane result in the reduction of transport membrane potential and the formation of ROS, which leads to the development of apoptosis [34]. In normal cells, ROS exists in equilibrium with antioxidant defenses, including superoxide dismutase (SOD2), catalase, peroxiredoxins, glutathione, and glutathione peroxidases (GPx). Increased concentrations of ROS is associated with oxidative stress and they are under the class of pro-oxidants found to cause damage to proteins, nucleic acid, lipids, and membranes, which lead to cell death [28]. Reactive nitrogen species are formed by the reaction between nitric oxide and ROS [35]. In this study, TA induced increase levels of ROS (Figure 4) may have contributed to the cell death that was observed in comet assay (Figure 6) and Hoechst assay (Figure 7). The increase in ROS led to the investigation of the effect of TA on antioxidants SOD2 and GPx. It was discovered that TA decreased SOD2 expression (Figure 8). The increased ROS and the downregulation of SOD2 was also observed in another study, where TA-induced apoptosis was associated with an increase in superoxide and an inhibition of SOD2 [9]. Glutathione peroxidase (GPx) plays an important role in hydrogen peroxide pro-oxidant effects. Tannic acid induced the downregulated expression of GPx (Figure 6), which validated that TA induced oxidative stress by increased ROS production and decreased expression of antioxidants to combat them. The decrease in RNS (Figure 5) would be caused by the optimum concentration of nitric oxide (NO), which has been reported to have a biphasic effect on oxidative stress when it is decomposed into nitrogen dioxide (NO2) and peroxynitrites (ONOO) [36]. Hence, further research on these RNS species could be needed to evaluate the participation of the individual species of RNS play with regards to TA action on HepG2 cells.

Reactive oxygen species also play an important role in the maintenance of the redox balance and in the activation of other signaling pathways and transcription factors, including Nuclear factor-like-2 (Nrf2) and the tumour suppressor p53 [28]. Nrf2 is a transcription factor that is expressed in a variety of cells that is upregulated by oxidative stress found in cells for the transcription of antioxidants [37]. Figure 8 showed a downregulated Nrf2 expression after treatment with TA, which further validated the TA-induced down-regulation of antioxidant proteins in the cells. The levels of ROS in cells were observed to be a key control of cell function [9]. Under physiological conditions, the presence of ROS activates p53, which plays a key role in controlling the stress responses by inducing cell cycle arrest (Figure 8B) in order to promote DNA repair and survival [38,39]. A downregulation of p53 (Figure 9) is a confounding result, since increased oxidative stress was observed in Figure 4 and Figure 5. However, p53 regulates cellular energy metabolism in addition to regulating antioxidant response [40]. Therefore, further studies are necessary for deciphering the mechanisms underlying p53 downregulation by TA.

## 4. Materials and Methods

### 4.1. Materials

The HepG2 cells were obtained from Highveld Biological (Johannesburg, South Africa). Tannic acid was purchased from Sigma Aldrich (Johannesburg, South Africa). All of the materials used in cell culture were purchased from Whitehead Scientific (Johannesburg, South Africa). The reagents used in western blot were purchased from Bio-Rad (Hercules, CA, USA) and all of the antibodies were purchased from Cell Signalling Technology (Anatech, Johannesburg, SA). All other reagents were obtained from Merck (Darmstadt, Germany), unless stated otherwise.

### 4.2. Cell Culture

Cryopreserved HepG2 cells were transferred to a 25 cm^3^ sterile flask, reconstituted in complete culture medium (CCM; Eagle’s minimum essential medium (EMEM), supplemented with 10% foetal calf serum, 1% L-glutamine, and 1% penicillin-streptomycin-fungizone), and incubated at 37 °C in a 5% CO_2_ atmosphere. The cells were maintained in sterile 25 cm^3^ cell culture flasks immersed in 5 mL of complete culture medium (CCM) until 80% confluency was reached. The cells were trypsinised and counted while using trypan blue.

### 4.3. Preparation of the TA Treatment

A stock solution of TA was prepared; 15 mg of TA was added to 7.5 mL of CCM in a sterile tube. The stock solution was then used in the different assays described below.

### 4.4. MTT Assay

The methylthiazol tetrazolium (MTT, Sigma, Johannesburg, South Africa) assay was conducted to determine the cytotoxicity and concentration that produced half the half maximal inhibitory concentration (IC_50_) for TA. HepG2 cells were seeded into a 96-well microtiter plate (Whitehead Scientific, Johannesburg, South Africa) at a concentration of 15,000 cells/well in triplicate; cells were permitted to attach overnight. The cells were incubated with a range of TA concentrations (0–58 μM) at 37 °C for 24 h. Thereafter, the treatments were removed, and the cells were incubated with 20 μL MTT salt solution (5 mg/mL in 0.1 M PBS) and 100 μL CCM at 37 °C for 4 h. The supernatants were then aspirated and 100 μL dimethyl sulfoxide (DMSO) was added to each well and then incubated at 37 °C for 1 h to solubilise the formazan crystals. The optical density (OD) was measured while using a plate reader Bio-Tek µQuant MQ×200 spectrophotometer (Bio-Tek μQuant, Winooski, VT, USA) at 570/690 nm. The percentage cell viability [(Average Absorbance of Treatment)/(Average Absorbance of Control) × 100] of the samples and a concentration-response curve was plotted using GraphPad Prism v5.0 software (GraphPad Software Inc., La Jolla, CA, USA) relative to the control. The concentration of TA that produced half of the maximum inhibition (IC_50_) was determined and used in the determination of IC_25_, which were both used for subsequent assays. Therefore, cytotoxicity testing was used as the basis for the selection of the initial concentrations of the TA needed to investigate the mechanism by which TA leads to cell death in HepG2 cells.

### 4.5. Luminometry

The cultured cells that had reached 80% confluency were treated with different concentrations of TA. Three flasks were used; for the control, 14.7 µM (IC_25_) and 29.4 µM (IC_50_), respectively. The flasks were incubated overnight at 37 °C, 5% CO_2_. The treated cells were then trypsinised and counted while using the trypan blue method, for the luminometric and comet assays.

#### 4.5.1. Adenosine Triphosphate (ATP) Quantification Assay

The levels of intracellular ATP are indicative of respiratory capacity and mitochondrial function. Intracellular ATP levels were quantified while using a CellTiter-Glo® assay. From the untreated control and treated cells, 50 μL of cell suspension (20,000 cells/well in 0.1M PBS) was seeded into a white, opaque 96-well luminometer plate in triplicate. Thereafter, 50 μL of ATP reagent was added into each well, followed by incubation of the plate in the dark for 30 min at room temperature (RT) to allow for the luciferin-luciferase reaction to occur. Luminescence, which is proportional to the level of intracellular ATP, was then detected while using a Modulus™ microplate luminometer (Turner Bio-systems, Sunnyvale, CA, USA) and the measurements were represented as a relative light unit (RLU).

#### 4.5.2. Measurement of Caspase Activity

The luminometric assay was used to assess the initiator caspases (caspase-8 and -9) and executioner caspases (caspase-3/-7) of apoptosis based on the cleavage of the luciferin substrate, which is specific to caspases; this reaction produces light that is directly proportional to caspase activity. The untreated control and treated cell suspension (20,000 cells/well in 50 μL 0.1 M PBS), respectively, were seeded into a white, opaque 96-well microtiter plate in triplicate. Caspase-Glo®-3/7, -8 and -9 reagents were reconstituted and 50 μL of each caspase reagent was added into each treatment well in triplicate. Thereafter, the plate was incubated in the dark (30 min, RT). Luminescence was detected while using a Modulus™ microplate luminometer and expressed as RLU. The data units were expressed as relative fold change for analysis.

### 4.6. Fluorescent Microscopy

Changes in the nucleus were investigated by the fluorescent microscopy (Olympus IXS1 inverted microscope, Tokyo, Japan).

#### 4.6.1. Single Cell Gel Electrophoresis (SCGE) Assay

The SCGE/comet assay derives its name from the pattern that is made by the fragmented DNA in single cells during electrophoresis. It is a highly sensitive technique that is used to detect DNA damage at a single eukaryotic cell level.

Frosted end microscope slides were prepared per control and treatments in duplicate; 800 µL of 2% LMPA was pipetted onto each slide, with a coverslip that is placed on the gel and solidified for 10 min at 4 °C, 400 µL of the second layer comprising 1 µL gel red, cell suspension (20,000 cells in 25 µL of PBS), and 1% LMPA (400 µL). When this layer set, the third layer containing 400 µL of 1% LMPA was pipetted and left to set for 10 min at 4 °C. Once all of the layers were set, coverslips were removed, and the slides were submerged in cold lysis buffer (100 mM EDTA, 2.5 M NaCl, 1% Triton X-100, 10% DMSO, and 10 mM Tris (pH 10)), so as to allow for the permeation of fragmented DNA out of the cell for an hour at 4 °C. The slides were removed from the lysis buffer and then submerged in the electrophoresis buffer (1 mM Na2, EDTA, and 300 mM NaOH; (pH 13)) for 20 min at RT to allow for equilibration prior to connecting an electric field for electrophoresis (25 V, 35 min). The slides were rinsed three times (5 min each) in 0.4 M Tris (pH 7.4) to neutralise the samples prior to replacement of coverslips. The slides were then viewed using a fluorescent microscope (Olympus IXS1 inverted microscope, Tokyo, Japan, excitation: 510–560 nm; emission 590 nm). Images of 50 cells and comets were captured in total from the slides and lengths of cells and comet tails were measured while using Soft imaging system (Life Science-©Olympus Soft Imaging Solutions v5, GmbH, Münster, Germany).

#### 4.6.2. Hoechst Assay

The cells were washed three times with PBS and trypsinised prior to being seeded in a 24-well microtiter plate (50,000 cells per well in 1 mL CCM) for 24 h at 37 °C. The cells were treated with the IC_25_, IC_50_, and the control, all in triplicate and incubated for 24 h at 37 °C. The treated cells were washed with PBS and then preserved with 10% paraformaldehyde (pH 7.4), followed by incubation for 5 min at RT. PFA was removed and cells were washed with 2 mL of PBS three times. After the last wash, 1mL of Hoechst 33342 (InvitrogenTM, Eugene, OR, USA) working solution (5 µg/mL in PBS) was added in each well. The plate was incubated at 37 °C for 15 min and then washed with PBS to remove any remaining Hoechst solution. This was then viewed under an Olympus IX51 inverted fluorescent microscope, while using 350 nm excitation and 450 nm emission filters.

### 4.7. Spectrophotometry

The control and treatment supernatants saved from previous assays were used for spectrophotometric (Bio-Tek μQuant, Winooski, VT, USA) assays.

#### 4.7.1. Thiobarbituric Acid Reactive Substances (TBARS) Assay

The TBARS assay was used to measure the lipid peroxidation end-product malondialdehyde, a reactive aldehyde that is produced by lipid peroxidation of polyunsaturated fatty acids. The supernatant from control and treatments (200 µL each) was added to four labelled test tubes according to control and treatments. Positive (199 µL of CCM + 1 µL of MDA) and negative (200 µL of CCM) controls were prepared and 200 µL of each added to appropriately labelled test tubes. The 7% concentration of phosphoric acid (H_3_PO_4_), 200 µL, was added to each test tube, followed by adding 400 µL of thiobarbituric acid/Butylated hydroxytoluene (TBA/BHT) solution to every sample except the blank (negative control), 1 M HCl was added to the negative control instead. The six test tubes were vortexed and 1 M HCl was added to all of the test tubes to adjust the pH to the acidic range. The samples were boiled in a water bath for 15 min. at 100 °C and then allowed to cool down to RT. Butanol (1500 µL) was then added to each tube and thoroughly vortexed. The tubes were allowed to stand until the two distinct phases became apparent. The samples (200 µL per well in duplicate) were then added to a 96-well plate and read via the Bio-Tek µQuant MQ×200 spectrophotometer at 532 nm with a reference wavelength of 600 nm. The average of three replicates was calculated and divided by the absorption coefficient, 156 mM^-1^ to determine the average concentration of MDA (µM).

#### 4.7.2. Nitric Oxide Synthase (NOS) Assay

The nitric oxide synthase (NOS) assay was used to detect NOS activity by using the spectrophotometry to measure the nitric oxide generated by the cells at the control and treatment concentrations of TA. Sodium nitrate standards were prepared at a concentration range of 0–200 µM. A 96-well microtiter plate was used to load 50 µL of each standard and 50 µL of each sample, all in duplicate. To these, 50 µL of Vanadium trichloride, 25 µL of sulphanilamide, and 50 µL of N-(1-naphthyl)-ethylenediamine dihydrochloride were consecutively added and incubated at 37 °C for 45 min. Absorbance was determined while using a Bio-Tek µQuant MQ×200 spectrophotometer at a wavelength of 540 nm and a reference wavelength of 690 nm. A standard curve was prepared and then used to determine the nitrate concentration, which was then analysed using GraphPad Prism v5.0 (GraphPad Software Inc., La Jolla, CA, USA).

### 4.8. Western Blotting

Western blotting was used to detect and quantify the expression of specific proteins in a homogenous sample. The proteins were represented as bands and the bandwidth is proportional to the quantity of the respective protein. Crude protein was isolated from cells using Cytobuster™ (Novagen, San Diego, CA, USA) supplemented with Roche (Mannheim, Germany) protease (05892791001) and phosphatase inhibitors (04906837001). Cytobuster reagent (300 µL) was added to treated flasks on ice for 15 min; the cells were scraped and then transferred to a 1.5 mL Eppendorf and incubated on ice for 10 min. The cell solution was then centrifuged at 10,000× *g*, 4 °C for 5 min. The supernatant was used for protein quantification while using the bicinchoninic acid (BCA) assay.

Samples and prepared bovine serum albumin (BSA) standards (0–1 mg/mL) (25 µL) were pipetted into a 96-well microtiter plate in duplicate. A 200 µL volume of BCA reagent (198 µL BCA: 4 µL CuSO_4_ per reaction) was added to each well and the plate was incubated at 37 °C for 30 min and the absorbance was read on a Bio-Tek µQuant MQ×200 spectrophotometer at 562 nm. A standard curve was plotted and the protein concentration of each sample was determined. The proteins were standardised to 1 mg/mL. Laemmli buffer [dH_2_O, 0.5 M Tris-HCl (pH 6.8), glycerol, 10% SDS, β-mercaptoethanol, 1% bromophenol blue] (200 µL) was added to each sample and then heated to 100 °C (5 min).

Standardised and denatured protein was loaded onto SDS polyacrylamide gel (10% resolving gel and 4% stacking gel) to separate the proteins. The gels were submerged in running buffer and subjected to an electric field for 90 min at 150 V. The gels were placed into transfer buffer (dH_2_O, Tris, glycine, methanol, pH 8.3) and then transferred onto a nitrocellulose membrane using the Transblot® TurboTM Transfer system (Bio-Rad, CA, USA). The nitrocellulose membranes were each blocked with 5 mL of 5% BSA in Tris-buffered saline (TTBS, 25 mM Tris (pH 7.5) 150 mM NaCl, 0.05% Tween 20) for 2 h, incubated with 5 mL of primary antibodies at 1:1000 dilution in 5% BSA (PARP-1 (9542), p53 (48818), GPx1 (3286), SOD2 (13141), and Nrf2 (12721)) for 1 h, and then left overnight at 4 °C. The primary antibody was removed and the membrane was washed five times with 10 mL of TTBS (10 min. per wash). The membrane was then incubated with 5 mL of secondary antibody (anti-rabbit IgG, 7074S (PARP-1, GPx1, SOD2 and Nrf2) or anti-mouse IgG, 7076 (p53)), respectively, in 1:2500 dilution in 5% BSA for 2 h. Following incubation, the membranes were washed with TTBS. Clarity Western ECL Substrate (Bio-Rad) (150 µL) was added to the membranes and the images were captured while using a gel documentation system Molecular imager® Chemidoc™ XRS+ and Bio-Rad imaging system.

Each membrane was then washed with 10 mL of distilled water and quenched with hydrogen peroxide (H_2_O_2_) (5 mL) at 37 °C for 30 min. The membranes were then washed for 1 min. with 10 mL dH_2_O and TTBS, consecutively. The stripped membranes were then blocked with 5 mL of 5% BSA for 2 h. Blocking solution was discarded and HRP-conjugated β-actin (Sigma, Johannesburg, South Africa), a house-keeping protein, was added to the membranes (5 mL; 1:1000) and then incubated for 1 h. The membranes were washed with TTBS following the incubation period. The Molecular imager® Chemidoc™ XRS+ and Bio-Rad imaging system were used to capture the image.

Analysis occurred by measuring the band intensity of each protein (Bio-Rad imaging system, CA, USA), the protein bands were normalised against β-actin.

### 4.9. Statistical Analysis

All statistical data and analysis were conducted while using the GraphPad Prism v5.0 software. The unpaired student t-test with Welch’s correction was performed to determine statistical significance. The one-way analysis of variance test (ANOVA) with post-test Bonferroni comparing all columns was used. A significance of *p* < 0.05 was reported.

### 4.10. Ethical Approval

Ethical approval was obtained from the Biomedical Research Ethics Administration (BE469/18).

## 5. Conclusions

This study focused on investigating the interaction of TA and specific liver cancer cells (HepG2) to provide insights into the mechanism by which TA leads to cell death. The results obtained showed that TA can induce apoptosis and oxidative stress in HepG2 cells after application of two concentrations determined via cytotoxicity assessment during 24 h of exposure. TA was found to induce apoptosis via extrinsic activation and intrinsic activation pathways, resulting in DNA fragmentation. TA also increased cellular ROS while reducing cellular antioxidant response, including superoxide dismutase (SOD2), and glutathione peroxidases (GPx) resulting in oxidative stress, which contributes to apoptosis occurring in a caspase-dependent and caspase-independent manner. Hence, TA was effective in causing the death of liver cancer cells. The effect of TA in normal cells still needs further research to evaluate the safety of TA in food and pharmaceutical products to prevent poisoning.
